# Repurposing Riociguat to Target a Novel Paracrine Nitric Oxide-TRPC6 Pathway to Prevent Podocyte Injury

**DOI:** 10.3390/ijms222212485

**Published:** 2021-11-19

**Authors:** Daan ‘t Hart, Jinhua Li, Johan van der Vlag, Tom Nijenhuis

**Affiliations:** 1 Department of Nephrology, Radboud Institute for Molecular Life Sciences (RIMLS), Radboud University Medical Center, 6525 GA Nijmegen, The Netherlands; daan.thart@radboudumc.nl (D.‘t.H.); johan.vandervlag@radboudumc.nl (J.v.d.V.); 2 Department of Anatomy and Developmental Biology, Monash University, Clayton, VIC 3800, Australia; jinhua.li@monash.edu

**Keywords:** Riociguat, nitric oxide, TRPC6, podocyte injury, focal segmental glomerulosclerosis

## Abstract

Increased expression and activity of the Ca^2+^ channel transient receptor potential channel 6 (TRPC6) is associated with focal segmental glomerulosclerosis, but therapeutic strategies to target TRPC6 are currently lacking. Nitric oxide (NO) is crucial for normal glomerular function and plays a protective role in preventing glomerular diseases. We investigated if NO prevents podocyte injury by inhibiting injurious TRPC6-mediated signaling in a soluble guanylate cyclase (sGC)-dependent manner and studied the therapeutic potential of the sGC stimulator Riociguat. Experiments were performed using human glomerular endothelial cells and podocytes. Podocyte injury was induced by Adriamycin incubation for 24 h, with or without the NO-donor S-Nitroso-N-acetyl-DL-penicillamine (SNAP), the sGC stimulator Riociguat or the TRPC6 inhibitor Larixyl Acetate (LA). NO and Riociguat stimulated cGMP synthesis in podocytes, decreased Adriamycin-induced TRPC6 expression, inhibited the Adriamycin-induced TRPC6-mediated Ca^2+^ influx and reduced podocyte injury. The protective effects of Riociguat and NO were blocked when sGC activity was inhibited with 1H-[1,2,4]Oxadiazolo[4,3-a]quinoxalin-1-one (ODQ) or when TRPC6 activity was inhibited by LA. Our data demonstrate a glomerular (e)NOS-NO-sGC-cGMP-TRPC6 pathway that prevents podocyte injury, which can be translated to future clinical use by, e.g., repurposing the market-approved drug Riociguat.

## 1. Introduction

The glomerular filtration barrier (GFB) is essential in the filtration of blood by restricting the passage of blood proteins in a charge and size-selective manner. The GFB consists of fenestrated glomerular endothelial cells (GEnC), the glomerular basement membrane (GBM) and podocytes [1]. Podocytes are visceral epithelial cells with interdigitating foot processes connected by slit diaphragm protein complexes that include transient receptor potential channel 6 (TRPC6), which is directly linked to the podocyte cytoskeleton. Importantly, podocyte injury is considered to play a key role in the pathogenesis of numerous glomerular diseases [2].

TRPC6 is known to be important for preserving normal renal filtration by adapting the podocyte cytoskeleton to changes in glomerular blood pressure [3]. However, the overexpression of TRPC6 is linked to acquired glomerular diseases, and causative gain-of-function mutations in TRPC6 cause hereditary forms of focal segmental glomerulosclerosis (FSGS) [4,5]. Moreover, TRPC6 knock-out in mice or rats confers protection to the development of experimental diabetic nephropathy, puromycin aminonucleoside (PAN)-induced nephrosis and Angiotensin II-induced glomerular hyperfiltration [6,7,8] Enhanced Ca^2+^ influx via TRPC6 has been shown to be detrimental to podocytes as manifested by cytoskeletal rearrangements and subsequent podocyte death [9,10,11].

Previous research from our lab showed that inhibition of phosphodiesterase type 5 (PDE5) by Sildenafil (Viagra) and the subsequent increased cyclic guanosine monophosphate (cGMP) levels protect against the development of glomerular injury by reducing the expression and activity of TRPC6 [12]. The elevated cGMP levels, via the inhibition of PDE5, stimulated protein kinase G (PKG), which in turn activated peroxisome proliferator-activated receptor γ (PPARγ). PPARγ was shown to bind to the promotor of TRPC6, thereby inhibiting TPRC6 promoter activity and the expression of TRPC6. In addition, several studies have shown that PKG can directly phosphorylate TRPC6 and inhibit TRPC6 activity [13,14]. Based on these findings, the induction of cGMP synthesis in podocytes, via endogenous factors or drugs other than Sildenafil, might protect against glomerular injury by preventing podocyte injury.

It is worthy of note that the endogenous paracrine factor nitric oxide (NO) activates its receptor soluble guanylyl cyclase (sGC), which converts guanosine monophosphate (GMP) into cGMP [15]. NO plays a crucial role in glomerular physiology and is required for normal glomerular and renal function [16,17]. Furthermore, NO acts as a protective factor in different kidney pathologies such as diabetic nephropathy and anti-GBM glomerulonephritis [18,19]. For example, C57BL/6J mice with a systemic eNOS deficiency developed proteinuria after Adriamycin treatment, whilst WT mice of this mouse strain are normally resistant to Adriamycin-induced nephropathy [20]. We therefore hypothesized that NO produced by GEnC serves as a protective paracrine factor for podocytes by reducing podocyte TRPC6 expression/activity via the stimulation of cGMP synthesis [21,22,23].

Notably, sGC is the molecular target for several drugs—for example, Riociguat—which are currently prescribed for non-renal disorders. We reason that these drugs might also hold therapeutic potential for glomerular diseases by interfering with injurious TRPC6 signaling in the podocyte. As Riociguat is already a market-approved drug, Riociguat might be swiftly repurposed for the treatment of glomerular diseases when the therapeutic potential of Riociguat for renal disorders is established [24,25,26].

In this study, we characterized the hypothetical paracrine (e)NOS-NO-sGC axis between GEnC and podocytes. Furthermore, we studied the therapeutic effect of NO and Riociguat on injured podocytes via the inhibition of deleterious TRPC6 signaling and found that NO reduces podocyte injury. In addition, Riociguat seems to have therapeutic potential for proteinuric glomerular diseases by interfering with injurious TRPC6 signaling.

## 2. Results

### 2.1. Glomerular Endothelial Cells and Podocytes Express eNOS and Produce NO

To evaluate the presence of the components for the suggested (e)NOS-NO-sGC-TRPC6 paracrine axis in GEnC and hPOD, we first addressed the expression of NO-producing enzymes in these cells. NO is produced by three different enzymes of the nitric oxide synthase (NOS) family; i.e., neuronal NOS (nNOS), inducible NOS (iNOS) and endothelial NOS (eNOS). nNOS could not be detected in either human glomerular endothelial cells (ciGEnC) or human podocytes (hPOD) at the mRNA level (Supplemental Figure S1A). However, both eNOS and iNOS mRNA could be observed in ciGEnC and hPOD (Supplemental Figure S1B,C). While eNOS protein expression could be detected at very low levels in hPOD and at high levels in ciGEnC (Supplemental Figure S1D), iNOS expression could not be detected at the protein level in ciGEnC nor hPOD, either unstimulated or after LPS stimulation (Supplemental Figure S1E).

**Figure 1:**
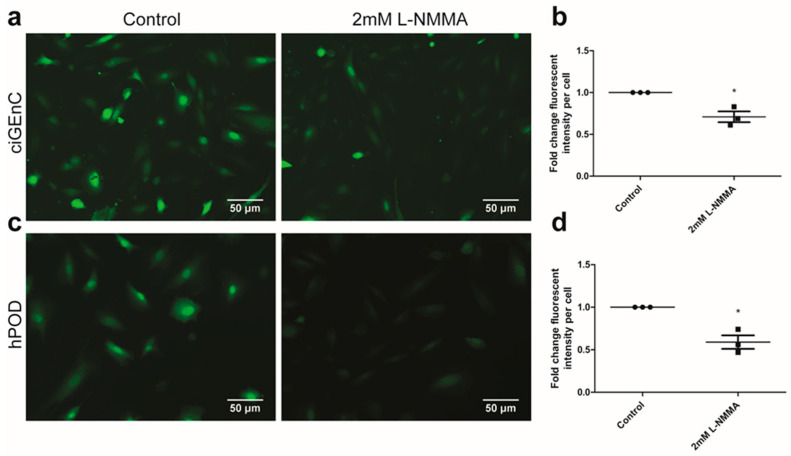
ciGEnC and hPOD synthesize NO. ciGEnC and hPOD were stained with 5 µM DAF-FM diacetate upon their respective differentiation period. (**a**) Representative images are shown from the ciGEnC left untreated (left panel) or treated with the NOS inhibitor L-NMMA (2 mM) (right panel). (**b**) Quantification of the fold change in the fluorescent intensity of the area * mean fluorescent intensity per cell for ciGEnC. (**c**) Representative images are shown from the hPOD left untreated (left panel) or treated with the NOS inhibitor L-NMMA (2 mM) (right panel). (**d**) Quantification of the fold change in the fluorescent intensity of the area * mean fluorescent intensity per cell for hPOD. * *p* < 0.05.

Next, we assessed NO production by ciGEnC (Figure 1a). To validate the specificity of the NO probe, ciGEnC were treated with the NOS inhibitor L-NMMA prior to the staining with the NO probe. Pre-treatment with L-NMMA reduced staining with the NO probe in ciGEnC (Figure 1a,b; *p* < 0.05). We also observed a positive staining with the NO probe in hPOD (Figure 1c). The specificity of the NO probe for hPOD was again confirmed by pre-treatment with L-NMMA (Figure 1c,d; *p* < 0.05). Glomerular eNOS protein expression in vivo primarily co-localized with cluster of differentiation 31 (CD31), but not with synaptopodin, suggesting glomerular endothelial eNOS protein expression but no podocyte expression (Supplemental Figure S2).

### 2.2. NO and Riociguat Increase cGMP Production and Downregulate TRPC6 Expression in hPOD

Next, we evaluated whether sGC subunits were expressed by ciGEnC and hPOD. We showed the expression of all four different subunits—i.e., sGCα1, sGCα2, sGCβ1 and sGCβ2—in hPOD, whereas only sGCα2 and sGCβ2 were expressed in ciGEnC (Supplemental Figure S3A–D).

**Figure 2:**
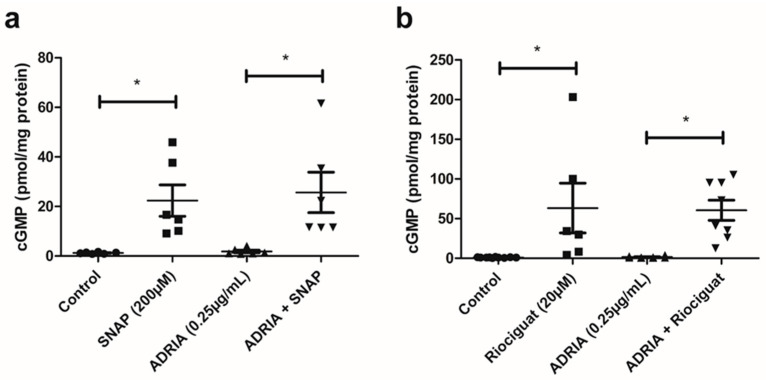
Riociguat and SNAP stimulate cGMP synthesis. (**a**) hPOD were incubated with 200 µM of the NO donor SNAP or (**b**) with 20 µM of the sGC stimulator Riociguat for 15 min and cGMP production was measured. Incubation was performed in the absence or presence of 0.25 µg/mL Adriamycin to mimic podocyte injury. * *p* < 0.05.

We investigated if NO and Riociguat are able to activate the sGC receptor in hPOD by measuring subsequent cGMP production. Exposure to the NO-donor SNAP resulted in ~20-fold increased cGMP production (Figure 2a; *p* < 0.05) in hPOD, whereas treatment with Riociguat caused a ~60-fold increase in cGMP synthesis (Figure 2b; *p* < 0.05). We subsequently evaluated whether NO and the sGC stimulator Riociguat activate sGC in Adriamycin-injured injured podocytes. Basal levels of cGMP production were not affected by Adriamycin (Figure 2a,b), and SNAP and Riociguat increased cGMP production in Adriamycin-injured podocytes to the same extent as in non-injured podocytes (Figure 2a,b; *p* < 0.05).

**Figure 3:**
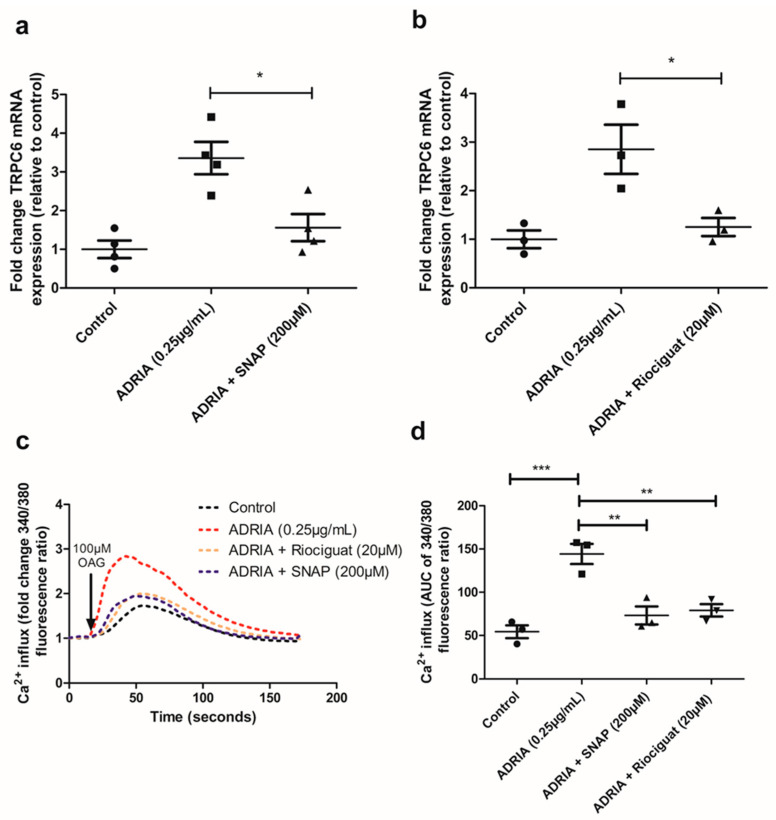
Riociguat and SNAP prevent Adriamycin-induced TRPC6 overexpression and activity in podocytes. hPOD were treated for 24 h with 0.25 µg/mL Adriamycin to induce podocyte injury. Podocytes were simultaneously treated with either 20 µM Riociguat or 200 µM SNAP. (**a**,**b**) RNA was isolated and TRPC6 mRNA expression was determined or (**c**) intracellular Ca^2+^ dynamics were determined using Fura-2 ratiometry upon stimulation with 100 µM OAG. (**d**) Area under the curve of the fold change in the 340/380 ratio of Fura-2 AM was calculated to quantify Ca^2+^ concentrations. * *p* < 0.05, ** *p* < 0.01, *** *p* < 0.001.

Thereafter, we addressed the effect of sGC activation by NO and Riociguat on TRPC6 expression in injured podocytes. Adriamycin-induced podocyte injury resulted in ~3-fold increased TRPC6 mRNA levels in hPOD (Figure 3a,b). Adriamycin-induced TRPC6 overexpression was almost completely prevented when podocytes were simultaneously incubated with the NO-donor SNAP (Figure 3a; *p* < 0.05) or the sGC stimulator Riociguat (Figure 3b; *p* < 0.05). Next, we investigated whether the SNAP and Riociguat-mediated downregulation of TRPC6 expression also translated into reduced TRPC6 activity. To measure Ca^2+^ influx, the hPOD cells were stimulated with 1-oleoyl-2-acetyl-sn-glycerolin (OAG), which activates and opens the TRPC6 channel [27]. Adriamycin treatment increased intracellular Ca^2+^ in hPOD after stimulation with OAG compared to control cells (Figure 3c,d; *p* < 0.001). Notably, co-treatment of Adriamycin with either SNAP or Riociguat decreased the Ca^2+^-concentration compared to hPOD treated with only Adriamycin (Figure 3c,d; *p* < 0.001). It is worthy of note that we could show that the Adriamycin-induced increase in Ca^2+-^-influx was TRPC6-dependent using the TRPC6-selective inhibitor Larixyl Acetate (Supplemental Figure S4A,B, *p* < 0.01) [28].

### 2.3. NO and Riociguat Downregulate Injury-Induced TRPC6 Expression via sGC Activation

**Figure 4:**
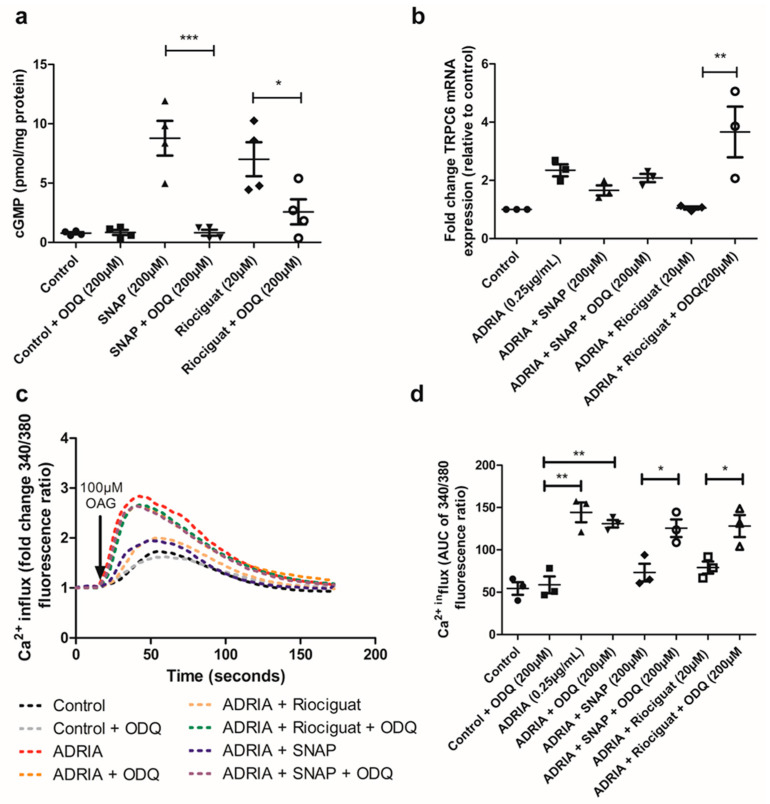
Inhibitory effects of Riociguat and SNAP are specifically mediated by sGC. (**a**) hPOD were stimulated with 20 µM Riociguat or 200 µM SNAP for 15 min in the absence or presence of 200 µM ODQ, and cGMP production was measured. (**b**) hPOD were treated for 24 h with 0.25 µg/mL Adriamycin to induce podocyte injury. Podocytes were simultaneously treated with either 20 µM Riociguat or 200 µM SNAP in the absence or presence of 200 µM ODQ, and TRPC6 mRNA expression was determined or (**c**) Ca^2+^ dynamics were investigated using Fura-2 upon stimulation with 100 µM OAG. (**d**) Area under the curve of the fold change in the 340/380 ratio of Fura-2 AM was calculated to quantify Ca^2+^ concentrations. * *p* < 0.05, ** *p* < 0.01, *** *p* < 0.001.

To prove that the effects of SNAP and Riociguat on cGMP production and Adriamycin-induced TRPC6 overexpression are specifically mediated via sGC, we evaluated the effect of the specific sGC inhibitor ODQ [29]. In the absence of sGC stimulation, exposure to ODQ did not alter baseline cGMP production in hPOD (Figure 4a). However, the SNAP and Riociguat-mediated increase in cGMP production was largely prevented by co-treatment with ODQ (Figure 4a; *p* < 0.001 and *p* < 0.05, respectively). Accordingly, ODQ also inhibited the protective effect of Riociguat on Adriamycin-induced TRPC6 overexpression (Figure 4b, *p* < 0.01), while ODQ treatment partially prevented the SNAP-mediated downregulation of TRPC6 expression during Adriamycin exposure (Figure 4b, *p* = 0.1). It is worthy of note that ODQ alone did not further increase Adriamycin-induced TRPC6 mRNA expression (Supplemental Figure S5). We subsequently investigated whether sGC inhibition by ODQ could prevent the effect of Riociguat and the NO-donor SNAP on Ca^2+^ influx in Adriamycin-injured hPOD. We found that, in the presence of ODQ, Riociguat treatment could not reduce the elevated Ca^2+^ influx in podocytes treated with Adriamycin (Figure 4c,d). In addition, although ODQ could only partially prevent the SNAP-mediated downregulation of TRPC6, ODQ completely blocked the protective effect of SNAP on Adriamycin-induced Ca^2+^ influx. In summary, both Riociguat and NO specifically inhibit Adriamycin-induced TRPC6 expression and activity via the activation of sGC.

### 2.4. sGC Activation Prevents PODOCYTE Injury via TRPC6 Inhibition

**Figure 5:**
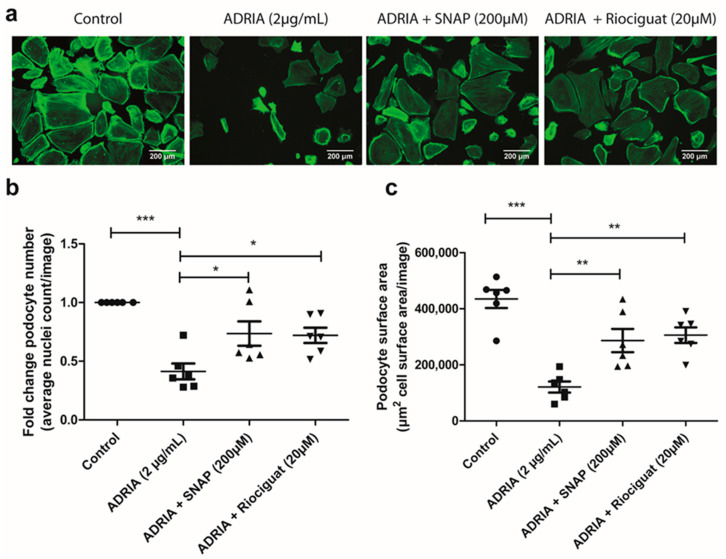
Riociguat and SNAP prevent Adriamycin-induced podocyte injury. hPOD were treated for 24 h with 2 µg/mL Adriamycin to induce podocyte injury. Podocytes were simultaneously treated with either 20 µM Riociguat or 200 µM SNAP. Podocytes were subsequently stained with Phalloidin-FITC and Hoechst 33342. (**a**) Representative pictures are shown for all conditions. Quantitative analyses in FIJI for (**b**) average cell count or (**c**) total cell surface area per image (C) * *p* < 0.05, ** *p* < 0.01, *** *p* < 0.001.

Podocyte injury and podocyte loss are important hallmarks of glomerular diseases. Therefore, we investigated whether the effect of NO and Riociguat on TRPC6 expression and activity in Adriamycin-injured podocytes also translated into an effect on podocyte injury. Exposure to Adriamycin caused a significant reduction in the podocyte number (Figure 5a,b) and a reduction in the total podocyte cell surface area (Figure 5a,c). sGC activation by either SNAP and Riociguat partially prevented podocyte injury (Figure 5a–c; *p* < 0.01 and *p* < 0.05, respectively).

**Figure 6:**
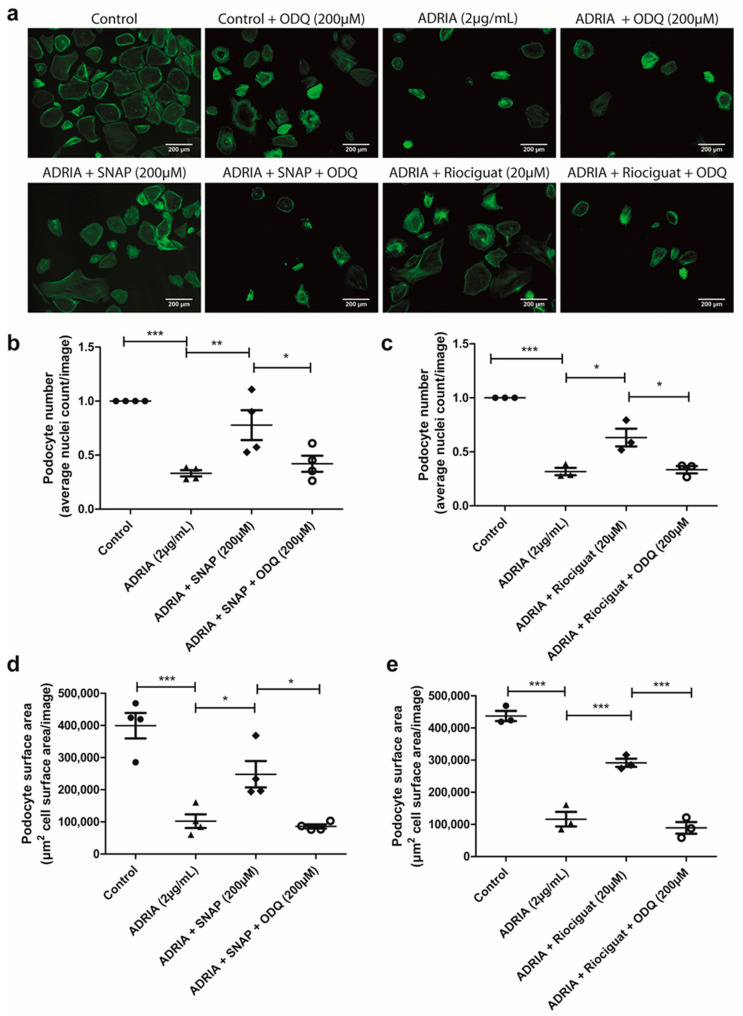
Riociguat and SNAP prevent Adriamycin-induced podocyte injury specifically by stimulating sGC. hPOD were treated for 24 h with 2 µg/mL Adriamycin to induce podocyte injury. Podocytes were simultaneously treated with either 20 µM Riociguat or 200 µM SNAP with or without 200 µM ODQ. Podocytes were subsequently stained with Phalloidin-FITC and Hoechst 33342. (**a**) Representative pictures are shown for all conditions. (**b**) Quantitative analyses in FIJI for average cell count for SNAP without or with ODQ and (**c**) Riociguat without or with ODQ treatment or total cell surface area per image for (**d**) SNAP without or with ODQ or (**e**) Riociguat without or with ODQ. * *p* < 0.05, ** *p* < 0.01, *** *p* < 0.001.

To show the importance of sGC in the aforementioned observations, we subsequently investigated whether sGC inhibition via ODQ could prevent the previously mentioned protective effects of SNAP and Riociguat on podocyte injury. When hPOD were co-incubated with ODQ, both Riociguat and SNAP could not prevent Adriamycin-induced podocyte cell injury (Figure 6a–e). We observed that ODQ affected podocyte viability in healthy control podocytes (Supplemental Figure S6). Importantly, upon inducing podocyte injury via Adriamycin, ODQ did not further increase podocyte injury (Supplemental Figure S6). These data exemplified that the inhibitory effect of ODQ on Riociguat- and SNAP-mediated protection against Adriamycin-induced podocyte injury was not caused by a damaging effect of ODQ itself.

**Figure 7:**
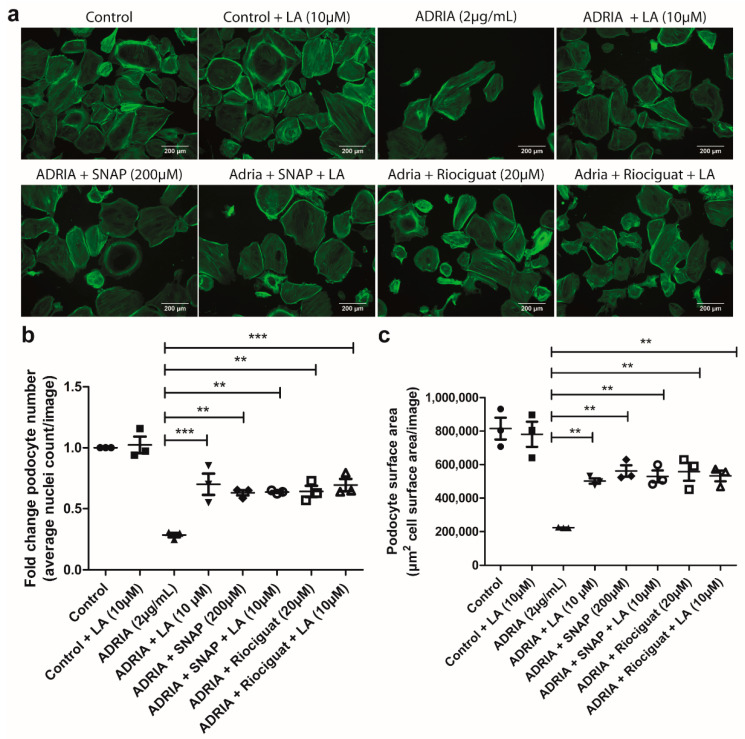
Riociguat and SNAP prevent Adriamycin-induced podocyte injury via inhibition of TRPC6. hPOD were treated for 24 h with 2 µg/mL Adriamycin to induce podocyte injury. Podocytes were simultaneously treated with 10 µM Larixyl Acetate (LA) with or without 20 µM Riociguat or 200 µM SNAP. Podocytes were subsequently stained with Phalloidin-FITC and Hoechst 33342. (**a**) Representative pictures are shown for all conditions. (**b**) Quantitative analyses in FIJI for average cell count for LA without or with Riociguat or SNAP and (**c**) total cell surface area per image for LA without or with Riociguat or SNAP. ** *p* < 0.01, *** *p* < 0.001.

Finally, we also investigated if the therapeutic effect of Riociguat and SNAP was specifically mediated via the inhibition of TRPC6 using the TRPC6-selective inhibitor LA [28]. We observed that LA partially prevented Adriamycin-induced podocyte injury (Figure 7a–c). When co-incubated with Riociguat or SNAP, LA did not provide additional protection against podocyte injury compared to treatment with Riociguat or SNAP alone. These results suggest that the protective effect of Riociguat and SNAP on Adriamycin-induced podocyte injury is specifically mediated via an interaction with TRPC6. In summary, we show that sGC activation in podocytes and subsequent TRPC6 inhibition via either NO or Riociguat prevents Adriamycin-induced podocyte injury.

### 2.5. NO Reduces Glomerular TRPC6 Overexpression In Vivo

**Figure 8:**
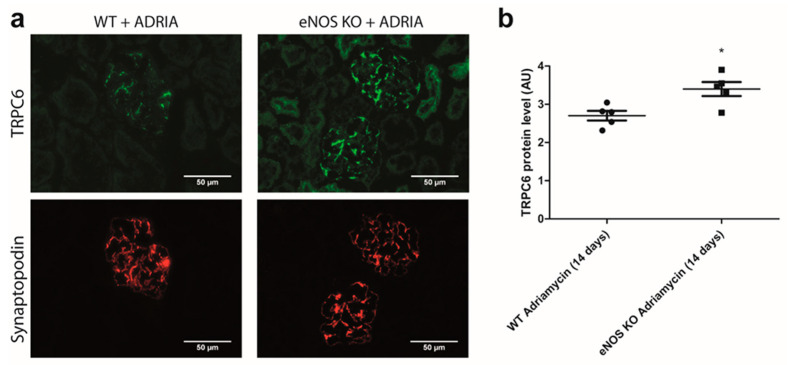
In mice, eNOS knockout enhances glomerular TRPC6 expression upon Adriamycin-induced nephropathy. Adriamycin nephropathy was induced in C57Bl/6J WT and eNOS KO mice, and mice were sacrificed after 2 weeks. (**a**) Representative pictures of the glomerular TRPC6 and synaptopodin expression in WT and eNOS KO mice. (**b**) Glomerular TRPC6 expression was determined via semi-quantitative analysis of immunofluorescent signal by scoring 25–30 glomeruli per animal on blinded sections performed independently by two investigators. N = 5 per group. * *p* < 0.05.

We also investigated if NO prevented glomerular TRPC6 overexpression during Adriamycin-induced nephropathy in vivo. Therefore, we measured glomerular TRPC6 expression in C57BL6/6J WT and C57BL6/6J eNOS KO mice after inducing nephropathy with Adriamycin. The C57BL6/6J mouse strain is normally resistant to the development of Adriamycin-induced nephropathy [30]. However, increased glomerular TRPC6 expression was observed in eNOS KO mice compared to WT mice (Figure 8a,b; *p* < 0.05). Thus, in line with our finding that sGC stimulation by NO decreases the expression and activity of TRPC6 during Adriamycin-mediated podocyte injury in vitro, the absence of NO leads to enhanced glomerular TRPC6 expression in a mouse strain normally resistant to Adriamycin-induced nephropathy.

## 3. Discussion

**Figure 9:**
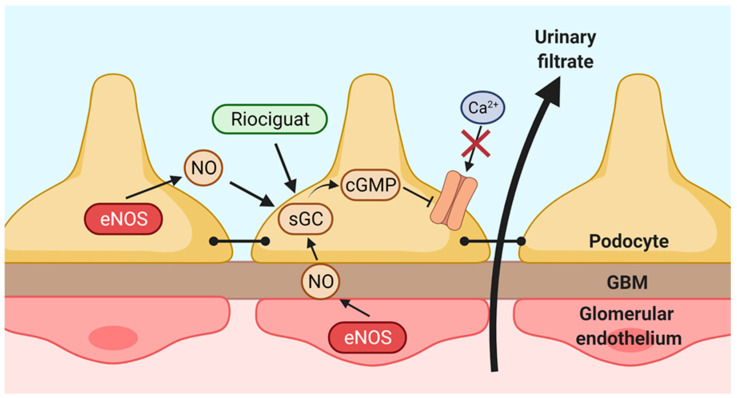
Schematic overview of the hypothetical paracrine (e)NOS-NO-sGC axis in the glomerulus. Nitric oxide (NO) is produced by endothelial nitric oxide synthase (eNOS) in glomerular endothelial cells (GEnC) and diffuses across the glomerular basement membrane (GBM) to the podocytes. In the podocyte, NO stimulates its receptor soluble guanylate cyclase (sGC) leading to increased cyclic guanosine monophosphate (cGMP) synthesis. The increased cGMP production inhibits the expression and activity of the Ca^2+^-channel transient receptor potential channel 6 (TRPC6), thereby inhibiting deleterious TRPC6 signaling and preventing downstream podocyte injury. NO might also be produced by podocytes themselves, thereby constituting a protective autocrine loop. In addition to NO, the sGC stimulator Riociguat, currently market approved for treatment of pulmonary arterial hypertension (PAH), might activate sGC to prevent podocyte injury.

The present study provides proof for the existence of a paracrine (e)NOS-NO-sGC axis between GEnC and podocytes (as depicted in Figure 9). In this paracrine axis, eNOS-derived NO from the glomerular endothelium activates sGC in podocytes, leading to enhanced cGMP synthesis. NO might also be produced by podocytes themselves, thereby forming an additional protective paracrine pathway. Elevated cGMP production inhibits TRPC6 expression, TRPC6-mediated Ca^2+^ influx and eventually prevents podocyte injury. Importantly, our data underpin the therapeutic potential of the sGC stimulator Riociguat in the context of podocyte injury, as we showed that Riociguat protects against experimental podocyte injury in an sGC-dependent manner.

In recent years, it has become increasingly evident that GEnC and podocytes are not merely two separate components of the glomerular filtration barrier. Instead, crosstalk between GEnC and podocytes is considered an important mechanism to maintain the integrity of the glomerular filtration barrier [31]. For example, podocyte-derived vascular endothelial growth factor (VEGF) is pivotal for the correct functioning of GEnC, and the absence of VEGF leads to glomerulopathies [32]. Moreover, the secretion of angiopoietins and endothelin-1 by podocytes is an important mechanism to prevent albuminuria [33,34]. We now demonstrate that NO might also be an important paracrine factor involved in the crosstalk between GEnC and podocytes by preventing TRPC6- and Ca^2+^ influx-mediated podocyte injury.

Reduced glomerular NO production is associated with several renal disorders; e.g., diabetic nephropathy and chronic kidney disease (CKD) [35,36,37,38,39]. Previous work has shown that eNOS deficiency aggravated the progression of experimental diabetic nephropathy by making podocytes more susceptible to injury [39]. Furthermore, a systemic eNOS knock-out compromised podocyte integrity in mice [38]. The precise mechanism by which NO protected podocytes against injury, however, remained largely elusive. In the current study, we demonstrate for the first time that NO stimulates the sGC receptor in podocytes, leading to elevated cGMP production, the inhibition of TRPC6 expression and activity and eventually protection against podocyte injury. We confirmed that this mechanism also acts in vivo, as eNOS KO mice showed higher glomerular TRPC6 protein expression levels than WT mice upon the induction of Adriamycin-induced nephropathy.

GEnC are well known to produce NO [22,40]. In this study, however, we provide evidence that podocytes can also produce NO. While we could not show eNOS expression by podocytes in healthy glomeruli, a recent study revealed that podocytes produce NO in response to angiotensin II stimulation [41]. Furthermore, NO production was suggested to be impaired in injured glomeruli, as, e.g., shown in hypertensive compared to control rats. Together with the protective effect of NO on podocyte integrity, we show in this study that podocyte-derived NO production might be an important autocrine protective mechanism of podocytes.

Various studies have highlighted the important role of TRPC6 in the pathogenesis of glomerular injury using TRPC6 knock-out animal models [6,7,8]. In most studies, TRPC6 knock-out animals showed markedly reduced glomerulosclerosis and podocyte foot effacement [6,7]. However, some studies have suggested that TRPC6 (over)expression could also protect podocytes against complement-mediated glomerular disease and that TRPC6 knock-out was not protective in a specific model of diabetic nephropathy [42,43]. Importantly, none of these studies used a tissue-specific or cell type-specific TRPC6 knock-out model. Loss of TRPC6 outside the kidney might activate compensatory mechanisms (e.g., alterations in blood pressure) which in turn affect kidney function. Therefore, to specifically investigate the role of TRPC6 in podocytes, future studies should focus on the development of podocyte-specific TRPC6 knock-out animal models. These animal models will provide a conclusive answer about the role of TRPC6 in the context of glomerular diseases and podocyte injury.

In addition to investigating the (e)NOS-NO-sGC axis, we also examined the therapeutic potential of the sGC stimulator Riociguat. We showed that Riociguat prevents Adriamycin-induced TRPC6 overexpression and activity by increasing sGC-mediated cGMP production. Increased cGMP production has been shown previously to exert renoprotective effects. For example, inhibition of PDE5 using Sildenafil (Viagra) or Tadalafil protected against the progression of various experimental models of kidney diseases [12,44]. In addition, a recent study showed that a combination treatment of the angiotensin II receptor antagonist Valsartan and the neprilysin inhibitor Sacubitril exerted improved renoprotective effects compared to treatment with Valsartan alone [45]. The therapeutic effect was assigned to increased urinary levels of atrial natriuretic peptide (ANP) in the combination group, a subsequent increased cGMP production and eventually decreased glomerular TRPC6 expression. The therapeutic potential of the sGC stimulators/activators Cinaciguat and IW-1973 on the development of, e.g., diabetic nephropathy and glomerulonephritis has also been previously shown [46,47,48]. For example, the sGC activator Cinaciguat protected diabetic rats against the development of proteinuria and podocyte injury whilst restoring the glomerular cGMP content [46]. Based on our findings, stimulating cGMP synthesis in podocytes via Riociguat might also be a promising and novel therapeutic option for the treatment of glomerular disorders. It is worthy of note that Riociguat was recently shown to reduce the progression of renal inflammation and kidney injury in an experimental mouse model of unilateral ureteral obstruction (UUO) [49].

Novel strategies to find new therapeutic options for glomerular injury are highly warranted because in the last decade only sodium glucose co-transporter 2 inhibitors have been approved as novel treatment options [50]. As an example, Canagliflozin was originally marketed for the treatment of type 2 diabetes but was recently shown to improve the renal outcome of patients with or without type 2 diabetes. Repurposing market-approved drugs such as Canagliflozin is an excellent example of an innovative strategy to rapidly find novel therapeutic options for the treatment of renal disorders. The sGC stimulator Riociguat is a market-approved drug and currently prescribed for patients suffering from pulmonary arterial hypertension and chronic thromboembolic pulmonary hypertension [25,26,51,52,53]. Riociguat might therefore swiftly be repurposed for the treatment of glomerular disorders if the therapeutic potential has been further clinically established.

In conclusion, we provide evidence that NO can serve as a paracrine and possibly also as an autocrine factor, which prevents podocyte injury via the sGC-mediated inhibition of deleterious TRPC6 signaling (Figure 9). Furthermore, the market-approved sGC stimulator Riociguat might be repurposed for the treatment of glomerular diseases, although its therapeutic potential should be further confirmed in vivo in clinical studies. Future studies should also focus on investigating the real-life paracrine interactions between GEnC and podocytes, for which the development of glomerulus-on-a-chip models will be instrumental.

## 4. Materials and Methods

### 4.1. Cell Culture

Previously characterized conditionally immortalized human podocytes (hPOD) and conditionally immortalized human glomerular endothelial cells (ciGEnC) were cultured as described previously [54,55]. Podocytes were exposed for 24 h to either 0.25 µg/mL or 2 µg/mL Adriamycin hydrochloride (Sigma-Aldrich, Schnelldorf, Germany) to induce podocyte injury, and/or simultaneously to 20 µM Riociguat (Selleckchem, Munich, Germany), 200 µM 1H-[1,2,4]Oxadiazolo[4,3-a]quinoxalin-1-one (ODQ, Sigma-Aldrich, Schnelldorf, Germany), 200 µM S-Nitroso-N-acetyl-DL-penicillamin (SNAP, NO donor, Abcam, Cambridge, UK) or 10 µM Larixyl Acetate (LA, Sigma-Aldich, Schnelldorf, Germany). hPOD and ciGEnC were stimulated for 24 h with 1 µg/mL LPS (serotype O111B4, Sigma-Aldrich, Schnelldorf, Germany) when protein samples were collected for the detection of iNOS protein expression on Western blot.

### 4.2. Animal Studies

WT C57BL6/6J mice (Monash Animal Services, Monash University, Australia) and eNOS-deficient mice in a C57BL6/6J background (Jackson Laboratories, Ben Harbor, ME, USA) were housed and bred at Monash Animal Services. Animal handling and experiments were approved by the Monash University Animal Ethics Committee and according to the “Australian Code of Practice for the Care and Use of Animal for Scientific Purposes”. Adriamycin-induced nephropathy was induced in 8 week old C57BL/6J WT and eNOS-KO mice by an intravenous injection of 10.5 mg/kg body weight of Adriamycin (Sigma-Aldrich, Milwaukee, WI, USA), as described previously [56]. Mice were sacrificed 14 days after induction of nephropathy. n = 5 mice per group.

### 4.3. RNA Isolation and Quantitative PCR Analysis

RNA was isolated from hPOD and ciGEnC using Trizol (Thermofisher, Scientific, Breda, The Netherlands) and 1 µg RNA was reverse-transcribed into cDNA using the Transcription First Strand cDNA synthesis kit (Roche, Woerden, The Netherlands) according to manufacturer’s instructions. Quantitative gene expression levels were determined by quantitative PCR using SYBR Green (Roche, Woerden, The Netherlands) on a CFX 96 C1000 Thermal Cycler (Bio-rad, Lunteren, The Netherlands) and were normalized to glyceraldehyde-3-phosphate dehydrogenase (GAPDH) levels using the delta-delta CT method. At least three independent experiments were performed, and each experiment consisted of at least three samples. All samples were measured in duplicate. Sequences of gene specific primers are listed in Table 1.

### 4.4. Nitric Oxide Detection

Production of NO in hPOD and ciGEnC was visualized using the NO-sensitive dye 4-Amino-5-Methylamino-2′,7′-Difluoroscein Diacetate (DAF-FM diacetate, Thermofisher Scientific, Breda, The Netherlands). Differentiated cells were incubated for 60 min with 10 µM DAF-FM diacetate in phenol red-free (Thermofisher Scientific, Breda, The Netherlands) and fetal bovine serum (FBS)-free medium at 37 °C. Cells were subsequently washed three times with Hank’s balanced salt solution (HBSS, Gibco, Breda, The Netherlands) to remove excess probe and rested for an additional 45 min to allow the complete de-esterification of the probe. Where indicated, cells were pre-treated with 2 mM of the NOS inhibitor NG-Methyl-l-arginine acetate salt (L-NMMA, Sigma-Aldrich, Schnelldorf, Germany). Cell nuclei were stained with 1 µg/mL Hoechst 33342 (Thermofisher Scientific, Breda, The Netherlands) for 5 min. Three independent experiments were performed, and each experiment consisted of at least two samples. At least five fluorescent images were captured using a Zeiss Imager (Carl Zeiss Microscopy, Jena, Germany). An M1 microscope was used for each sample immediately upon completing the staining. FIJI (1.47v, National Institutes of Health, Bethesda, Rockville, MD, USA) was used for the quantification of fluorescent intensity stainings. The experiment was repeated in triplicate.

### 4.5. cGMP ELISA

cGMP levels in hPOD were measured using the direct cGMP EIA kit (Enzo Life Sciences, Breda, The Netherlands) according to the manufacturer’s protocol. Prior to stimulation, medium was replaced by Dutch modified RPMI 1640 medium without supplements (Thermofisher Scientific, Breda, The Netherlands), except 1% Glutamax (Thermofisher Scientific, Breda, The Netherlands). The hPOD cells were subsequently incubated for 1 h with 1mM of the non-selective cGMP phosphodiesterase inhibitor 3-isobutyl-1-methylxanthine (IBMX, Sigma-Aldrich, Schnelldorf, Germany). Upon 1 h of incubation with IBMX, cGMP synthesis was stimulated by adding 20 µM Riociguat or 200 µM SNAP, with or without 200 µM ODQ for 15 min in the presence of IBMX. The reaction was stopped by the addition of 0.1 M HCl for 30 min at room temperature (RT). The acetylated protocol for preparing samples was followed according to manufactures instructions and samples were incubated for 24 h at 4 °C in the ELISA plate The protein content of cell extracts was determined using the bicinchoninic acid assay (Sigma-Aldrich, Schnelldorf, Germany) to correct for differences in cell density. The experiment was repeated in sextuplicate, and samples were measured in duplicate.

### 4.6. Immunohistochemistry

Cells were fixed with 2% paraformaldehyde (PFA, Sigma-Aldrich, Breda, The Netherlands) for 10 min. Subsequently, cells were permeabilized for 10 min with a 0.3% Triton X-100 solution (Thermofisher Scientific, Breda, The Netherlands) in 1× PBS and blocked for 30 min with blocking solution consisting of 2% BSA (Sigma-Aldrich, Breda, The Netherlands), 2% FBS (Sigma-Aldrich, Breda, The Netherlands) and 0.2% fish gelatin (Sigma-Aldrich, Breda, The Netherlands). The actin cytoskeleton of podocytes was visualized by staining with Phalloidin-FITC (1:200 dilution, Thermofisher Scientific, Breda, The Netherlands) in 1% BSA and 0.1% sodium azide solution for 45 min. Cell nuclei were visualized by staining with 1 µg/mL Hoechst 33342 (Thermofisher Scientific, Breda, The Netherlands) for 5 min. At least three independent experiments were performed, and each experiment consisted of three samples. For each sample, at least three fluorescent images were captured with a Leica DMI6000B microscope (Leica, Wetzlar, Germany). FIJI (1.47v, National Institutes of Health, Bethesda, Rockville, MD, USA) was used for counting the cell nuclei and the calculation of the surface area of the cells.

Immunofluorescent staining of eNOS, TRPC6, Synaptopodin and cluster of differentiation 31 (CD31) was performed on 2 µm thick cryosections of normal human kidney, as described previously. Primary antibodies included anti-eNOS (AB5589, Abcam, Cambridge, UK, 1:50 dilution), anti-TRPC6 (ACC017, Alomone Laboratories, Jerusalem, Israel, 1:200 dilution), anti-Synaptopodin (65194, Progen, Heidelberg, Germany, 1:5 dilution) and anti-cluster of differentiation 31 (CD31) (555444, BD Pharmingen, San Jose, CA, USA, 1:100 dilution). Glomerular TRPC6 expression was scored semi-quantitatively on a scale from 0–10 (0 = no staining, 5 = 50% staining and 10 = 100% staining) by two researchers, who scored 25–30 glomeruli per animal on blinded sections. Cell-type specific eNOS expression in glomeruli was determined using a double staining with synaptopodin and CD31. To validate the specificity of all antibodies, a negative control condition was included during every staining. At the negative control condition, the tissue was only incubated with the secondary antibody.

### 4.7. Intracellular Ca^2+^ Measurements by Fura-2 Ratiometry

Intracellular Ca^2+^ concentration was measured using the fluorescent Ca^2+^ indicator Fura-2 acetoxymethyl ester (Fura-2 AM, Thermofisher Scientific, Breda, The Netherlands). hPOD were stained with 2.5 µM Fura-2 AM for 30 min at 37 °C in HBSS (1.26 mM Ca^2+^) supplemented with 4.45 mM d-Glucose (Sigma-Aldrich, Schnelldorf, Germany) and 10 mM 4-(2-hydroxyethyl)-1-piperazineethanesulfonic acid (HEPES) (Sigma-Aldrich, Schnelldorf, Germany). Subsequently, cells were washed three times with HBSS supplemented with 4.45 mM d-Glucose and 10 mM HEPES. During experiments with the TRPC6 inhibitor LA, cells were exposed to 10 µM LA (Sigma-Aldrich, Schnelldorf, Germany) 5 min prior to imaging. Subsequently, cells were stimulated with 100 µM 1-oleoyl-2-acetyl-sn-glycerolin (OAG) (Sigma-Aldrich, Schnelldorf, Germany) and intracellular Ca^2+^ was determined by measuring the fluorescence at 340 and 380 nm using a BD Pathway 855 microscope (BD Biosciences, San Jose, CA, USA) every 2.5 s for 3 min. The fluorescent ratio of 340/380 nm of individual hPOD cells was calculated in FIJI (1.47v, National Institutes of Health, Bethesda, Rockville, MD, USA). Five individual cells per well were analyzed and at least five wells per condition were imaged. The experiment was repeated in triplicate. Data are presented as the fold change in the 340/380 nm ratio compared to the 340/380 nm ratio prior to OAG stimulation. The area under the curve (AUC) of the 340/380 nm ratio was calculated in GraphPad Prism version 5.03 (GraphPad Software, Inc., San Diego, CA, USA).

### 4.8. Western Blot Analyses

Protein samples were prepared according to previously published protocols using ELB buffer (250 mM NaCl (Sigma-Aldrich, Schnelldorf, Germany), 0.1% NP-40 (Sigma-Aldrich, Schnelldorf, Germany), 50 mM HEPES (pH = 7) and 5 mM EDTA(Sigma-Aldrich, Schnelldorf, Germany)) [57]. The protein content of cell extracts was determined using the bicinchoninic acid assay (Sigma-Aldrich, Schnelldorf, Germany) to ensure equal sample loading. For each condition, 20 µg of total protein was resolved by 8% SDS-PAGE, and proteins were subsequently transferred to nitrocellulose membranes (Bio-rad, Lunteren, The Netherlands). Membranes were blocked O/N at 4 °C with a 5% non-fat dry milk in PBS with 0.05% Tween-20 (PBS-T0.05) (Bio-rad, Lunteren, The Netherlands) and incubated for 1 h at RT with primary antibodies against eNOS (1:1000 dilution, abcam, Cambridge, UK, ab5589) and iNOS (1:500, abcam, Cambridge, UK, ab178945) in 1% non-fat dry milk in PBS-T0.05. Membranes were subsequently incubated for 1 h at RT with an HRP-conjugated donkey α-rabbit secondary antibody (1:2500 dilution, Jackson Immuno Reserarch, Cambridge, UK). Protein bands were visualized using SuperSignalTM West Pico PLUS Chemiluminescent (Thermofisher Scientific, Breda, The Netherlands) on a ChemidocTM XRS+ (Bio-rad, Lunteren, The Netherlands).

### 4.9. Statistical Analyses

Data are presented as mean ± SEM. Statistical analysis was conducted by a two-tailed Student’s t-test when comparing glomerular TRPC6 protein expression (n = 5 mice per group). A one-way ANOVA, followed by a Tukey’s multiple comparison test, was used to determine differences in the cGMP production, TRPC6 mRNA expression, AUC of Fura-2 live imaging experiments, podocyte count and podocyte surface area between experimental conditions. For the statistical analysis of fold changes in fluorescent intensity, a paired t-test was applied. All statistical analysis were performed using GraphPad Prism version 5.03 (GraphPad Software, Inc., San Diego, CA, USA). A *p*-value of <0.05 was considered statistically significant.

## Figures and Tables

**Table 1:** List of gene-specific primers used in this study.

Gene Name	Gene Symbol	Forward Sequence	Reverse Sequence
GAPDH	GAPDH	AGATGGTGATGGGATTTC	TCCTCGCATGATTGTCACCC
TRPC6	TRPC6	AATGCTCCCAGAAATGGCAC	CCTCCACTGAACCCTGGAAA
nNOS	NOS1	CTTCAAGAAGCTAGCAGAAGCTGT	ACAAGGACCAGAGTTTCATGTTC
iNOS	NOS2	ACAACAAATTCAGGTACGCTGTG	TCTGATCAATGTCATGAGCAAAGG
eNOS	NOS3	CCAGCTAGCCAAAGTCACCAT	GTCTCGGAGCCATACAGGATT
sGCα1	GUCY1A1	GCTCTTCTCAGACATCGTTGGG	ATAGGCATCGCCAATGGTCTCC
sGCα2	GUCY1A2	GCAGACTCTCAAGAGGACACTG	GTTGGAGTGGTCTGCATAGGAG
sGCβ1	GUCY1B1	GGAAATCCTCACTGACAGGCTAC	CAGCTCATTGGCAACAGACGGA
sGCβ2	GUCY1B2	TCTGCAACGCTTTTCCTTTCC	CGACTTTGCCATGCTACAGG

## Data Availability

The data presented in this study are openly available in the Open Science Framework at DOI:10.17605/OSF.IO/63REW.

## Supplementary Materials

The following are available online at https://www.mdpi.com/article/10.3390/ijms222212485/s1.
