# Supplementary material for: Repurposing Riociguat to Target a Novel Paracrine Nitric Oxide-TRPC6 Pathway to Prevent Podocyte Injury
Source: Int J Mol Sci. 2021 Nov 19;22(22):12485. doi: 10.3390/ijms222212485 (PMC8621407; doi:10.3390/ijms222212485)
Supplement: Supplementary file 1 [file ijms-22-12485-s001.zip › ijms-1458259-supplementary.pdf]

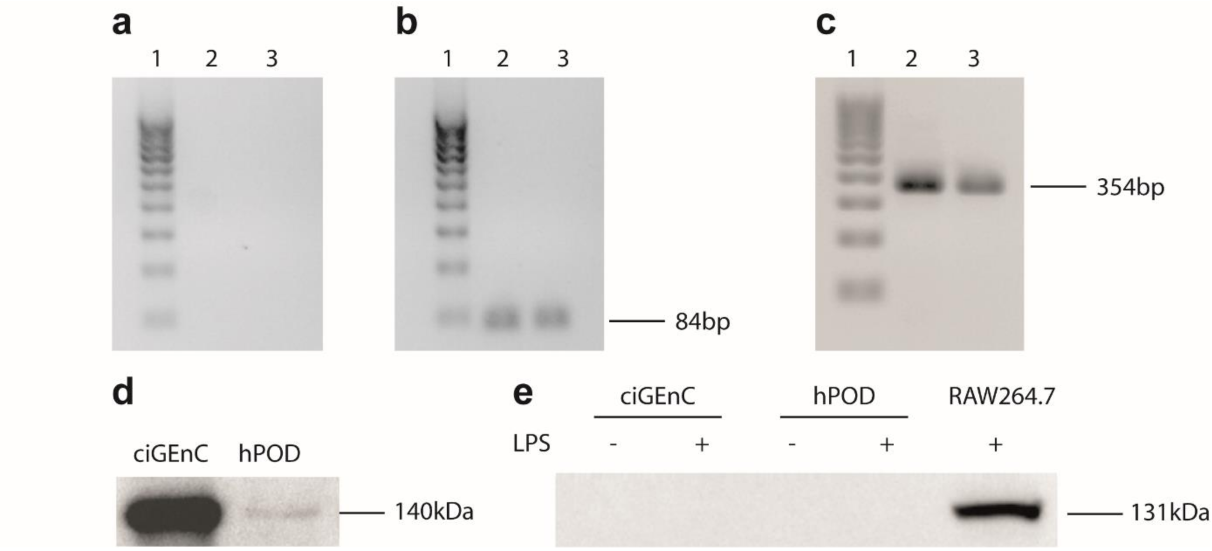

Figure S1. ciGEnC and hPOD express the NO producing enzyme eNOS. RNA and protein samples from hPOD and ciGEnC were collected upon their respective differentiation period. Representative agarose gel analysis of PCR products for (**a**) nNOS (**b**), iNOS and (**c**) eNOS. Lane 2 contains ciGEnC RNA and Lane 3 hPOD RNA. (**d**) Protein expression of eNOS visualized in unstimulated ciGEnC and hPOD using Western blot. (**e**) Western blot for iNOS on protein samples from ciGEnC and hPOD either left unstimulated or treated for 24hrs with 1µg/mL LPS. Protein lysate from RAW264.7 stimulated with 1µg/mL LPS was used as positive control.

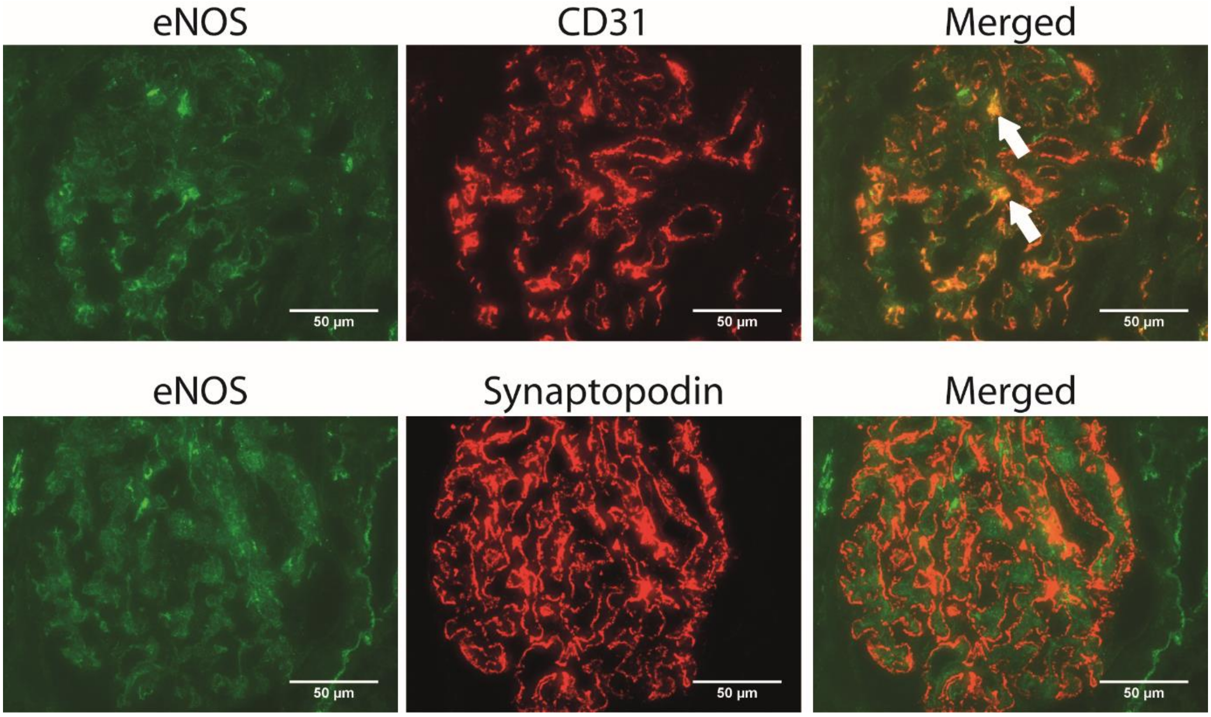

Figure S2. GEnC express eNOS *in vivo*. Normal human kidney tissue was double-stained for eNOS expression and CD31 or Synaptopodin to localize cell type-specific eNOS expression. Arrows indicate co-localization of eNOS and CD31 staining.

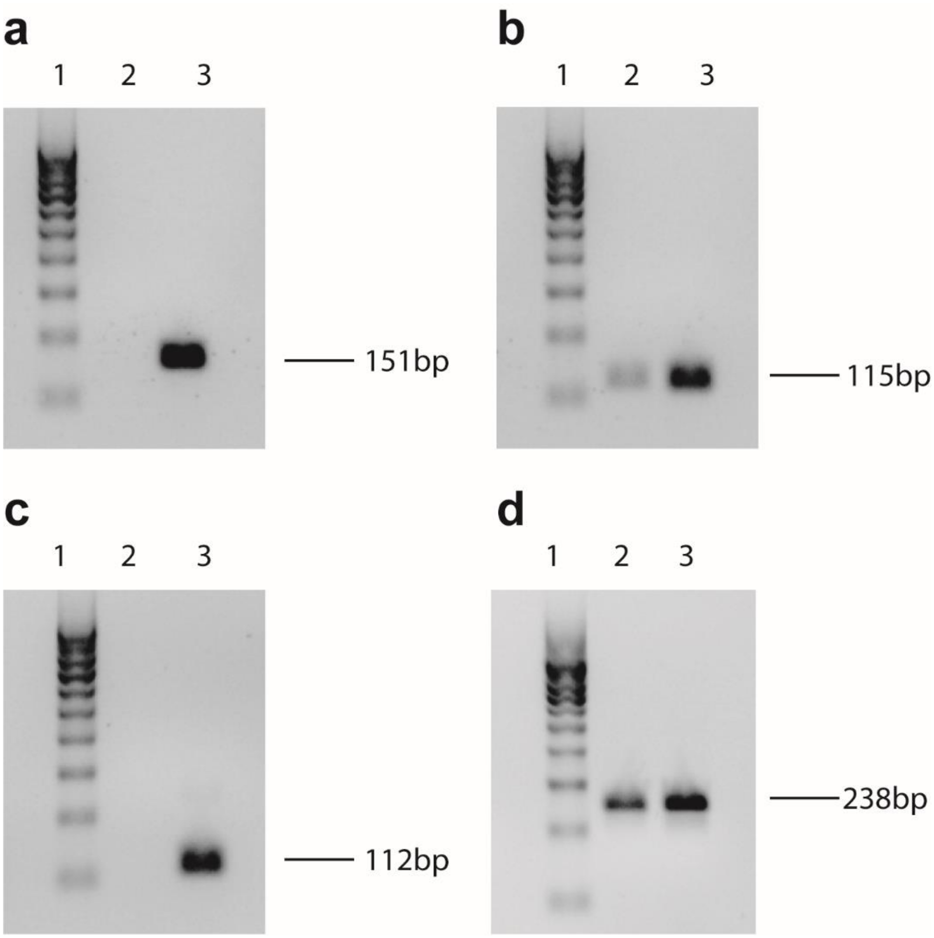

Figure S3. ciGEnC and hPOD express different sGC subunits. RNA samples from hPOD and ciGEnC were collected upon their respective differentiation period. Representative agarose gel analysis of PCR products for (**a**) sGCα1 (**b**), sGCα2 (**c**), sGCβ1 and (**d**) sGCβ2. Lane 2 contains ciGEnC RNA and Lane 3 hPOD RNA.

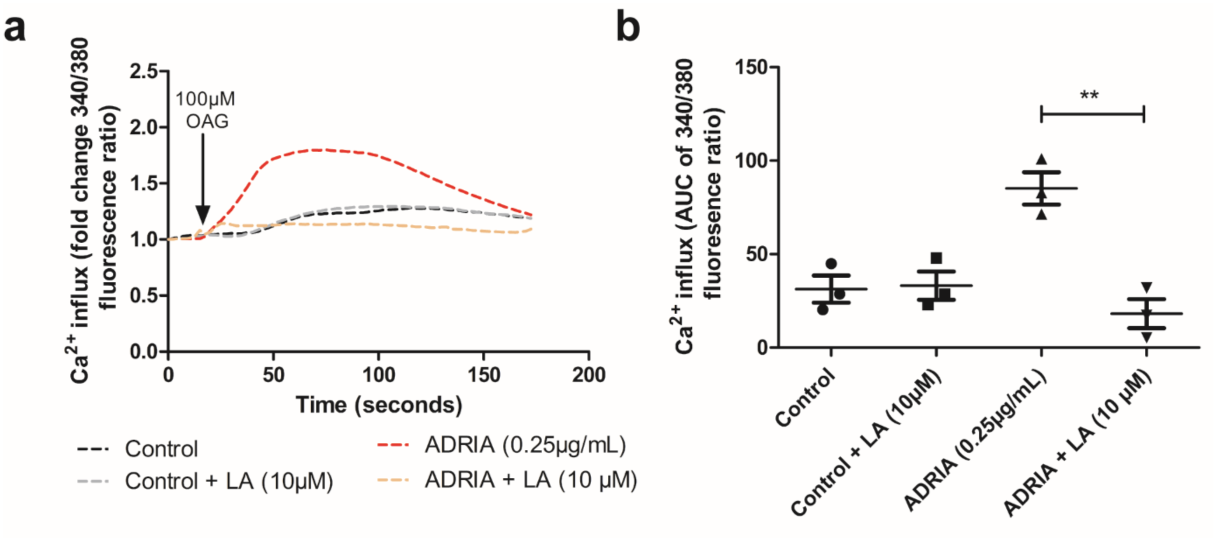

Figure S4. The Adriamycin-induced increase in Ca^{2+} influx is specifically mediated via TRPC6. hPOD were treated for 24h with 0.25µg/mL Adriamycin to induce podocyte injury. (**a**) Ca^{2+} dynamics were investigated using Fura-2 upon stimulation with 100µM OAG. 5 minutes prior to stimulation were exposed to the TRPC6 inhibitor Larixyl Acetate (LA). Area under the curve of the fold change in the 340/380 ratio of Fura-2 AM was calculated to quantify Ca^{2+} concentrations. ** $P < 0.01$

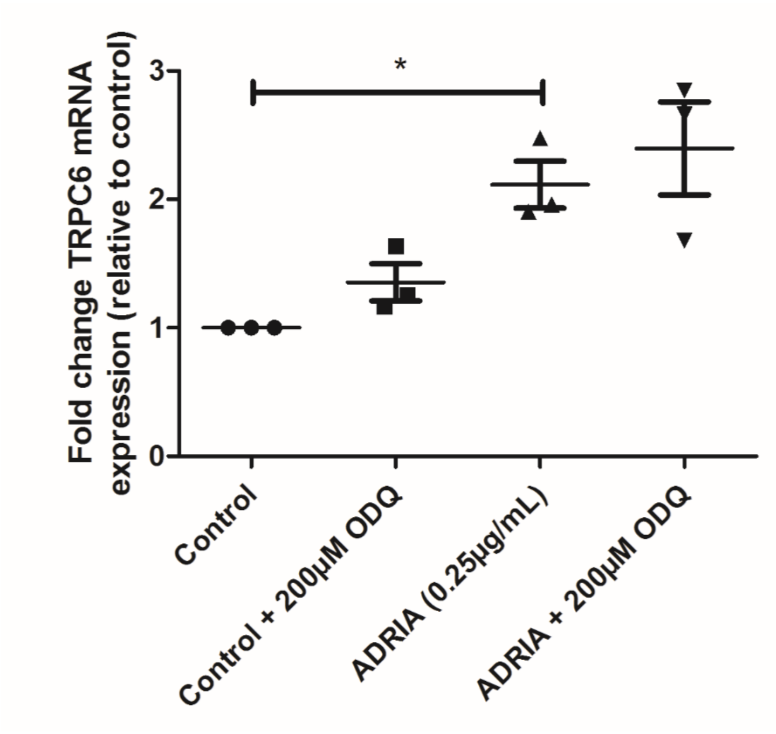

Figure S5. ODQ does not increase TRPC6 expression during Adriamycin treatment. hPOD were treated for 24hrs with 0.25µg/mL Adriamycin to induce podocyte injury. Podocytes were simultaneously treated with either 200µM ODQ. After 24hrs, RNA was isolated and TRPC6 mRNA expression was determined. * $P < 0.05$

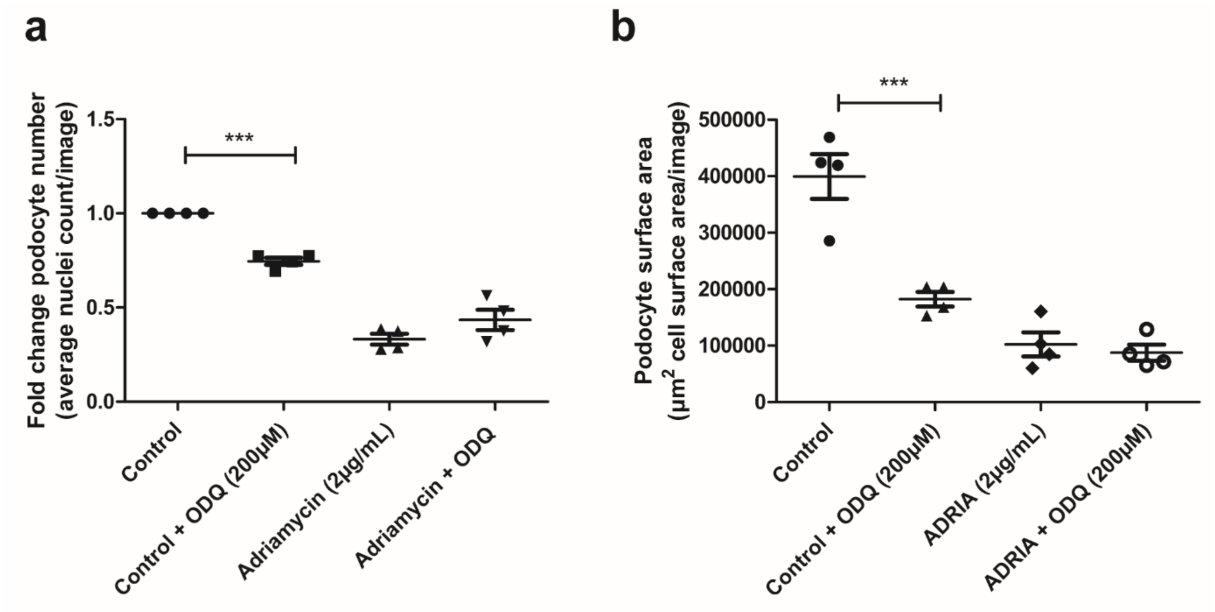

Figure S6. ODQ does not increase podocyte injury during Adriamycin treatment. hPOD were treated for 24hrs with 0.25µg/mL Adriamycin to induce podocyte injury. Podocytes were simultaneously treated with either 200µM ODQ. Podocytes were subsequently stained with Phalloidin-FITC and Hoechst 33342. (**a**) Quantitative analyses in FIJI for average cell count and (**b**) total cell surface area per image. *** $P < 0.001$.
